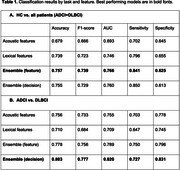# Automatic classification of patients in the AD spectrum from those in the DLB spectrum using speech

**DOI:** 10.1002/alz70856_103016

**Published:** 2025-12-25

**Authors:** Sunghye Cho, Taehwan Kim, Sung‐Woo Kim, Myungjin Ko, Junhyuk Louis Kwak, Mark Y Liberman, Min Seok Baek

**Affiliations:** ^1^ Linguistic Data Consortium, University of Pennsylvania, Philadelphia, PA, USA; ^2^ Silvia Health Inc., Seoul, Korea, Republic of (South); ^3^ Wonju Severance Christian Hospital, Yonsei University Wonju College of Medicine, Wonju, Korea, Republic of (South)

## Abstract

**Background:**

Early clinical symptoms of both Alzheimer's disease (AD) and dementia with Lewy body (DLB) include memory problems and difficulties in judgement and planning. With similar clinical symptoms, it is challenging to screen patients with AD at scale while excluding those with DLB. This study proposes automatic classification systems that utilize both text and acoustic features to automatically distinguish patients with AD from those with DLB.

**Method:**

Patients with AD pathology, including both AD and mild cognitive impairment (MCI) due to AD (ADCI; *n* = 55, 35 females, age=75.69±6.79y, education=9.78±4.49y), those with DLB or MCI due to DLB (DLBCI; *n* = 28, 11 females, age=77.93±5.79y, education=9.14±4.49y), and healthy elderly speakers (HC; *n* = 55, 29 females, age=72.51±6.35y, education=9.87±4.19y) completed a 5‐minute speech collection protocol that included one picture description task, two semantic fluency tasks, three letter fluency tasks, and one sustained phonation task. Amyloid positivity was confirmed with visual assessment of 18F‐Florbetaben PET, and all patients in the ADCI group exhibited amyloid positivity. We employed a state‐of‐the‐art large language model (KLUE‐RoBERTa‐base) to extract token‐level embeddings from concatenated texts of 1 picture description and five fluency tasks, which were mean‐pooled to generate participant‐level embedding features (*n* = 768). OpenSMILE (the EMOBASE configuration) was employed to extract acoustic features (*n* = 988) from the sustained phonation task. We applied Principal Component Analysis to reduce feature dimensions in each feature set respectively, and we experimented with two fusion methods (data‐level vs. decision‐level) in training Multilayer Perceptron classifiers for two binary tasks: HC vs. all patients (ADCI+DLBCI) and ADCI vs. DLBCI. The models were validated using five‐fold cross validation.

**Result:**

Table 1 summarizes all results. The classifier for distinguishing HC from all patients showed the best performance (accuracy=75.7%, AUC=0.77) when the text embeddings and the acoustic features were fused at the feature level. Text‐only or acoustic‐only models showed lower performances compared to the fused models. For distinguishing between ADCI and DLBCI, the model trained with decision‐level fusion showed the best performance (accuracy=80.3%, AUC=0.82), followed by the feature‐level fusion model (accuracy=77.8%, AUC=0.79).

**Conclusion:**

Our study demonstrates that the automatic classification of ADCI and DLBCI using speech is highly promising for large‐scale screening of patients for clinical trials and interventions.